# In-Plane Mechanical Properties of a Tetra-Missing Rib Symmetry Honeycomb

**DOI:** 10.3390/ma19030553

**Published:** 2026-01-30

**Authors:** Xiaolin Deng, Qi Lu, Zhenzhen Cai, Xinping Zhang

**Affiliations:** 1 School of Electronics and Information Engineering, Wuzhou University, Wuzhou 543002, China; caizhenzhen0827@163.com (Z.C.); 1827837018@163.com (X.Z.); 2 School of Mechanical and Electrical Engineering, Guilin University of Electronic Technology, Guilin 541004, China; luqi_3@163.com

**Keywords:** in-plane, tetra-missing rib honeycomb, symmetrical design, mechanical properties

## Abstract

Tetra-missing rib honeycombs (TMRHs), characterized by monoclinic geometry, exhibit high elastic stiffness but suffer from poor deformation stability and reduced axial load-bearing capacity, which limit their applicability in energy-absorbing and load-sensitive engineering structures. To address these inherent drawbacks, this study proposes two symmetry-enhanced tetra-missing rib honeycomb configurations through overall axisymmetric design and subunit-level symmetric optimization. A finite element model was established in Abaqus/Explicit and validated against quasi-static compression experiments, demonstrating good agreement in deformation modes and mechanical responses. Systematic numerical investigations were then conducted to compare the mechanical properties and deformation behaviors of three honeycomb layouts, including the conventional TMRH and the proposed symmetric designs. Furthermore, the effects of impact velocity on mechanical performance were examined to evaluate the dynamic response characteristics of the structures. Finally, the influence of subunit angle parameters on the stiffness, energy absorption, and deformation stability of the tetra-missing rib honeycombs was comprehensively analyzed. The results provide insight into the role of symmetry and geometric parameters in improving the mechanical performance of TMRH-based structures and offer guidance for the design of high-performance auxetic honeycombs.

## 1. Introduction

Honeycomb materials, as a representative class of multifunctional lightweight materials, exhibit outstanding mechanical characteristics, including high specific stiffness and strength, energy absorption capacity [1,2,3], thermal insulation, and shock-mitigation ability [4,5,6]. Over the past several decades, these materials have garnered significant research interest [7,8]. Advances in manufacturing techniques have facilitated their widespread adoption in aerospace, automotive, marine, packaging, and other industrial sectors [9,10]. In these application domains, honeycomb and thin-walled cellular structures are primarily employed for crashworthiness, impact mitigation, and weight reduction, where the required mechanical properties strongly depend on specific service conditions and functional objectives. For instance, aerospace and railway applications emphasize high specific energy absorption and structural stability under dynamic loading, whereas automotive and maritime structures often require a balance between peak load control, progressive collapse behavior, and manufacturability. Consequently, tailoring the deformation modes and mechanical responses of honeycomb structures to meet application-specific requirements has become a central research focus. In particular, additive manufacturing (AM) technologies have emerged as a powerful and versatile approach for fabricating honeycomb and cellular sandwich structures with complex geometries and tailored architectures [11,12]. Compared with conventional manufacturing methods, AM enables precise control over microstructural topology, relative density, and geometric parameters, thereby facilitating the realization of advanced auxetic and architected metamaterials. Fundamental studies have demonstrated the feasibility of AM-enabled lightweight cellular structures for energy absorption, load-bearing, and multifunctional integration, providing a solid technological foundation for both traditional and innovative honeycomb applications [12,13,14]. Increasing attention has been paid to green and sustainable materials driven by environmental regulations and circular-economy strategies, particularly within the European Union. The European Commission has emphasized recyclability and sustainability as key requirements for advanced engineering materials. In this context, aluminum alloys are widely regarded as attractive candidates for lightweight structures due to their excellent recyclability and well-established recycling infrastructure. However, polymer-based cellular materials often suffer from environmental concerns related to end-of-life disposal. Notably, biodegradable and recyclable polymers such as polylactic acid (PLA) offer a promising alternative, enabling the fabrication of complex honeycomb architectures while mitigating environmental impact. Therefore, the exploration of lightweight honeycomb structures made from recyclable metallic materials or sustainable polymers is of both scientific and practical significance.

Recent studies have focused on the mechanical response of honeycomb structures under various loading scenarios, such as tension [15,16], compression [17,18], buckling [19], shear [20], and fatigue [21,22,23,24]. The deformation behavior and mechanical performance of honeycomb structures are strongly influenced by their microstructural topology, and unique properties can be achieved by optimizing the microstructural arrangement [25,26,27]. In this context, several typical honeycomb configurations have been designed and improved [28,29,30]. A particularly intriguing phenomenon is the negative Poisson’s ratio (NPR) effect. Conventional hexagonal honeycombs typically exhibit lateral expansion under compressive loading, corresponding to a positive Poisson’s ratio. The physical origin and existence of such counterintuitive behavior were first demonstrated in pioneering studies by Lakes [31], who reported the first man-made auxetic foam, and by Wojciechowski, who revealed thermodynamically stable planar model systems exhibiting a negative Poisson’s ratio prior to the formal introduction of the term “auxetics” [32,33]. Such materials and structures are commonly referred to as auxetics, following the definition later proposed by Evans [34]. Xiao et al. [35] investigated the mechanical performance of honeycomb structures with negative Poisson’s ratios under low, medium, and high strain rates, revealing that the NPR effect can amplify inertial effects during dynamic compression. The auxetic effect has been observed across multiple length scales, ranging from macroscopic cellular structures to micro- and nanoscale architectures. Beyond the continuum scale, similar auxetic mechanisms have also been demonstrated at the atomic level. Narojczyk et al. [36] showed that a two-dimensional system composed of asymmetric trimer molecules can exhibit auxetic behavior, thereby confirming that the double arrowhead-type geometry can be realized even in molecular-scale systems.

Numerous novel auxetic configurations have been developed, including re-entrant structures [37,38,39], chiral structures [40,41,42], arrowhead structures [43,44], and rotating polygonal structures [45], with their in-plane mechanical properties receiving extensive attention. Re-entrant honeycomb structures, originally introduced by Gibson et al. [46]. Zhang et al. [47,48] conducted a systematic investigation of the yielding and tensile behavior of re-entrant hexagonal honeycombs from two principal perspectives: examining the influence of wall thickness and initial cell angle through finite element simulations and employing two material models. Their study discussed how various geometric and material parameters affect the mechanical response of re-entrant cellular structures. It should be noted that the anti-fourfold chiral family of cellular architectures encompasses a wide range of geometrical realizations, and numerous structures with distinct topological features have been reported in the literature [49,50,51]. Dudek et al. [52,53] presented a mathematical analysis of the mechanical and thermal expansion behaviors of single-mode metamaterials composed of rotationally rigid triangles and proposed a novel mechanical metamaterial incorporating both two-dimensional and three-dimensional microlayers with competing substructures, capable of exhibiting a wide range of unusual auxetic behaviors. Leveraging the superior mechanical performance of double-arrow honeycomb (DAH) materials and their broad applicability in aerospace, marine, and automotive fields, Jiang et al. [54] introduced double-arc walls into conventional DAH unit cells to enhance material strength and energy absorption. Based on arc wall placement, they proposed three circular double-arrow honeycomb (CDAH) configurations—upper circular (u-type), lower circular (l-type), and full circular (f-type)—and investigated their mechanical characteristics in detail. Further studies by Zhao et al. [55], Wang et al. [56], and Lan et al. [57] explored the arrowhead structures. Hu et al. [58] analyzed the mechanical behavior of anti-trichiral honeycombs under large quasi-static compressive deformations through combined experimental and theoretical approaches, revealing that cell collapse is governed by both ligament rotation around plastic hinges and cylinder rotation. Additionally, Hu et al. [59] employed numerical and theoretical methods to investigate the NPR effect of re-entrant anti-trichiral honeycombs under large deformations, ultimately focusing on their mechanical performance. The study of chiral structures remains an active research area: Tabacu et al. [60] used experimental and computational approaches to examine the compressive behavior of anti-tetra-chiral honeycombs fabricated from laser-cut tubes, while Zhang et al. [61] also contributed further findings on this topic.

As a distinct variant of tetra-chiral honeycombs, the tetra-missing rib honeycomb (TMRH) has attracted increasing research attention due to its unconventional mechanical behavior. Gaspar et al. [62] investigated the deformation mechanisms and Poisson’s ratio characteristics of this structure. Zhu et al. [63] were among the first to introduce a tetra-missing-rib-type configuration, which can be regarded as an early form of the TMRH concept, and developed analytical mechanical models based on a simplified energy approach under infinitesimal deformations, primarily focusing on elastic stiffness tailoring. Their study demonstrated that, compared with conventional non-auxetic lattices, the proposed configuration exhibits enhanced stiffness at low relative densities. Furthermore, Zhu et al. [63] proposed a modified tetra-missing rib metamaterial resembling traditional tetra-chiral honeycombs, which retains monoclinic behavior—characterized by coupling between shear and axial deformation—and exhibits improved elastic stiffness owing to this monoclinic symmetry.

Building upon these foundational studies, the present work focuses on the systematic investigation of the in-plane mechanical response and deformation mechanisms of TMRH structures under different layouts, loading conditions, and geometric parameters, which have not been comprehensively addressed in Ref. [63]. To further enhance the mechanical performance of tetra-missing rib honeycombs, Zhang et al. [64] incorporated soft materials into the plastic framework, creating plastic two-phase composites that improved structural behavior. Liu et al. [65] proposed an NPR anti-terachiral design with fully flexible beams; its in-plane mechanical properties were theoretically evaluated using an energy-based method and subsequently validated through finite-element simulations. In this paper, the deformation characteristics of the tetra-missing rib structure are summarized, along with its unique monoclinic characteristics. To reduce the influence of monoclinic characteristics on the in-plane mechanical properties of the tetra-missing rib structure, we introduce the concept of symmetry into the design and improve the in-plane mechanical properties of the structure as much as possible on the premise of improving the unique deformation of the structure itself. After combining the design concept of symmetry, the tetra-missing rib structure forms two new structures. The first is the tetra-missing rib structure with axisymmetric features, and the second is a new type of tetra-missing rib structure. Subsequently, quasi-static experiments are conducted on the tetra-missing rib structure to verify the finite element numerical model, and according to the structural parameters of the tetra-missing rib honeycomb, the mechanical properties and mechanical behavior of three honeycomb structures, including the tetra-missing rib honeycomb, are studied. It should be noted that we previously carried out quasi-static compression experiments on tetra-missing rib honeycomb, as well as analyzed the effect of the cell number of the structure on the structure by using the finite element method, and explored the dynamic Poisson’s ratio of three honeycombs. However, the relationship between velocity, angle, and energy absorption of the structure is still unclear, and the comparison of the mechanical properties of the tetra-missing rib honeycomb with other structures has not been carried out further. Therefore, this thesis proposes to further develop the comparative analyses of angle and velocity, as well as the performance of tetra-missing rib honeycomb with other structures, on the basis of the previous studies [66], to further elaborate the performance characteristics of tetra-missing rib honeycomb structures.

## 2. Structure and Method

### 2.1. Structure Design

**Figure 1:**
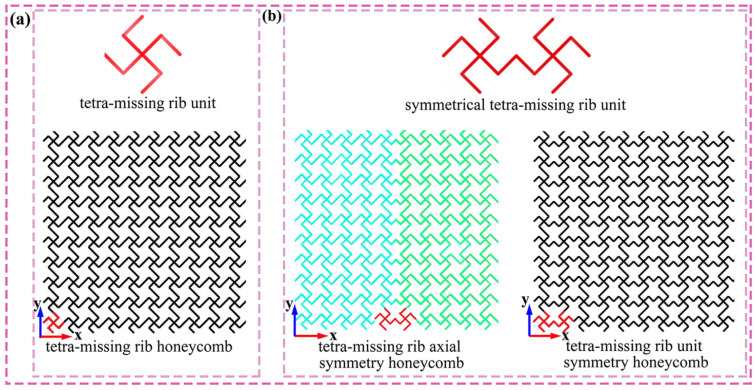
Structural design: (**a**) tetra-missing rib unit; (**b**) honeycomb structures with two distinct symmetry forms.

**Figure 2:**
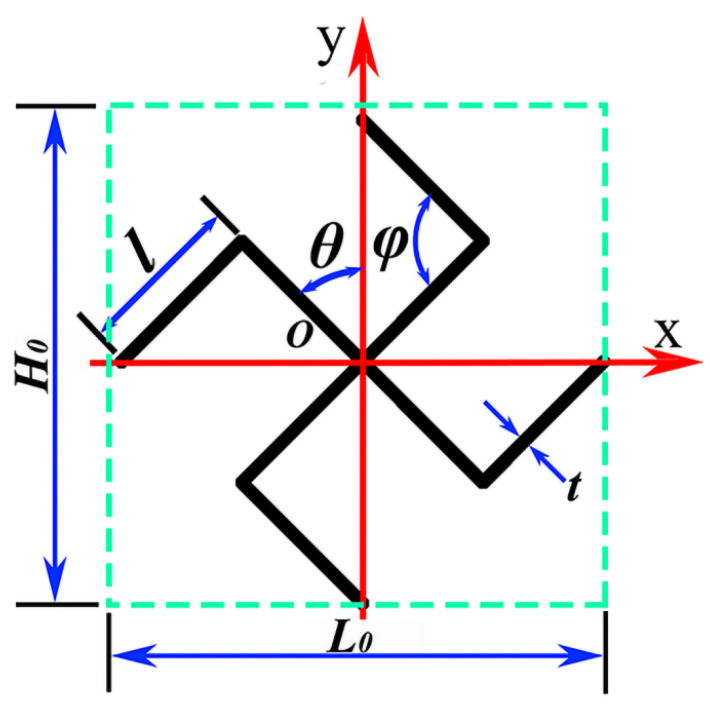
Tetra-missing rib unit and size parameters.

The novel structure proposed in this study is illustrated in Figure 1. Figure 1a depicts a conventional tetra-missing rib honeycomb, which serves as the basis for the design. The axisymmetric unit is constructed by connecting a tetra-missing rib subunit with its mirrored counterpart, as shown in Figure 1b. Two honeycomb configurations arise from different layout strategies applied to this symmetrical tetra-missing rib unit: the tetra-missing rib axial-symmetry honeycomb (TMRAH) and the tetra-missing-rib unit-symmetry honeycomb (TMRUH). The geometric parameters of the tetra-missing rib subunit are presented in Figure 2, where *H*_0_ = *L*_0_, *l* = 5 mm, *θ* denotes the angle between the tetra-missing rib unit and the y-axis, and φ represents the angle between the two ribs of the subunit. Following this parameterization, the honeycomb structures are systematically denoted as TMRH-*θ*-*φ*, TMRAH-*θ*-*φ*, and TMRUH-*θ*-*φ*. It should be emphasized that TMRAH is not introduced as a fundamentally new topology, but as a symmetry-enhanced configuration obtained by a mirrored assembly of the original TMRH. Although the absolute compressive strength and stiffness of honeycomb structures depend on the base material, previous studies [67,68] on aluminum and composite honeycombs have shown that deformation modes, collapse stability, and energy absorption efficiency are predominantly governed by cell topology and geometric configuration. In this regard, the symmetric design strategies proposed in this study are expected to be applicable to honeycomb cores fabricated from metallic and composite materials.

### 2.2. Mechanical Performance Index

To assess the performance of the unit cell, several commonly used evaluation metrics are employed. In these expressions, *σ* and *ε* denote the nominal stress and nominal strain of the honeycomb, respectively, which are defined as follows:

(1) $$\sigma =\frac{F}{Lb}$$

(2) $$\varepsilon =\frac{\Delta H}{H}$$

Here, *F* represents the impact force, while *L* and *b* denote the width and out-of-plane thickness of the TMRH, respectively, with *b* = 20 mm in this study. Δ*H* corresponds to the impact displacement, and *H* indicates the initial height of the structure.

The energy absorption (EA) is defined as the area under the stress–strain curve up to the densification strain, and it can be calculated using the following expression [69]:

(3) $$EA=\int_{0}^{\varepsilon_{d}}\sigma \left(\varepsilon \right)d\left(\varepsilon \right)$$

The plateau stress $\sigma_{pl}$ represents the average stress between yielding and densification during compression, and it can be determined using the following expression [70]:

(4) $$\sigma_{pl}=\frac{1}{\varepsilon_{d}-\varepsilon_{y}}\int_{\varepsilon_{y}}^{\varepsilon_{d}}\sigma (\varepsilon )d\varepsilon$$

In this expression, $\varepsilon_{y}$ denotes the yield strain, $\varepsilon_{d}$ is the densification strain, and σ(ε) represents the nominal stress corresponding to the nominal strain *ε*.

$\sigma_{m}$ represents the mean value of the nominal stress during the impact process, and the corresponding expression is as follows:

(5) $$\sigma_{m}=\frac{1}{\varepsilon_{d}}\int_{0}^{\varepsilon_{d}}\;\sigma (\varepsilon )d\varepsilon$$

The specific energy absorption (SEA) represents the energy absorbed per unit mass, and it can be calculated using the following expression [71]:

(6) $$SEA=\frac{EA}{m}=\frac{\int_{0}^{\varepsilon_{d}}\;\sigma (\varepsilon )d\varepsilon }{m}$$

where *m* is the mass of the honeycomb.

The peak stress $\sigma_{peak}$ corresponds to the first maximum on the stress–strain curve of the honeycomb structure.

## 3. Finite Element Model and Quasi-Static Experiment

### 3.1. Finite Element Model Construction

**Figure 3:**
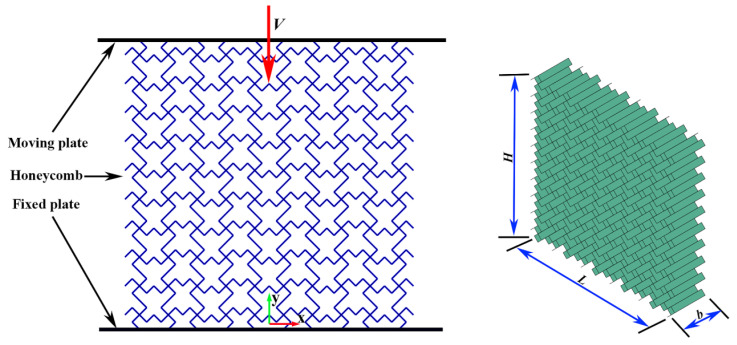
Finite element numerical model.

In this study, numerical simulations were conducted using Abaqus/Explicit (Abaqus/CAE, 6.14-1). The finite element model is illustrated in Figure 3. The honeycomb structure is positioned between two rigid plates: the lower plate is bound to the honeycomb and fully constrained, with all degrees of freedom of the rigid plates restricted, while the upper plate impacts the honeycomb at a constant velocity. In the simulation, the rigid plates are modeled as analytical rigid bodies, and the TMRH is represented using S4R shell elements with five integration points through the thickness. The mesh size for the finite element model is set to 0.8 mm. To prevent interpenetration between the honeycomb and the rigid plates, a general contact algorithm is applied to handle self-contact between the TMRH and the plates. The tangential contact behavior is defined by a friction coefficient of 0.2 [72], while the normal behavior is set as “hard” contact. The honeycomb is composed of aluminum alloy AA6061-O [73], characterized by a Young’s modulus E = 70 GPa, density ρ = 2700 kg/m^3^, Poisson’s ratio *v* = 0.3, and yield stress $\sigma_{y}$ = 130 MPa. Material behavior is modeled using an elastic–perfectly plastic constitutive law within Abaqus/Explicit.

### 3.2. Quasi-Static Experimental Verification

**Figure 4:**
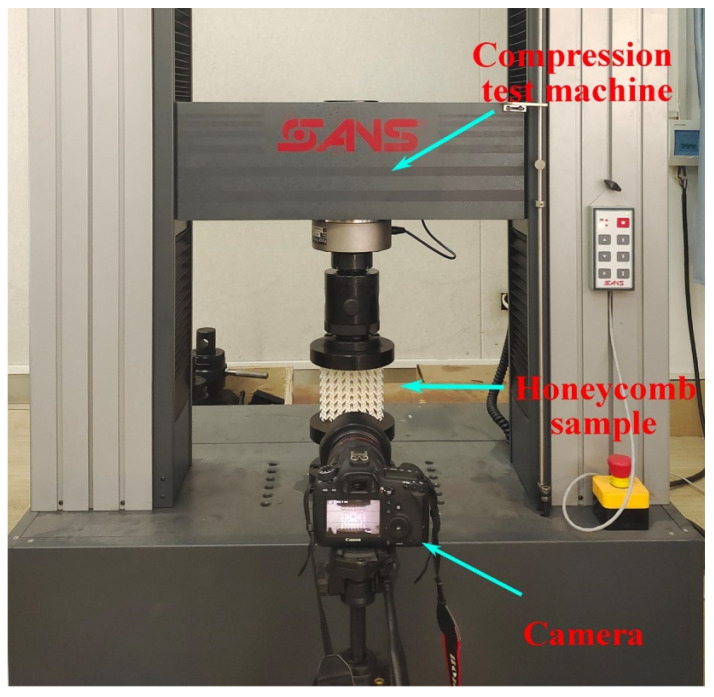
Quasi-static compression experiment.

**Figure 5:**
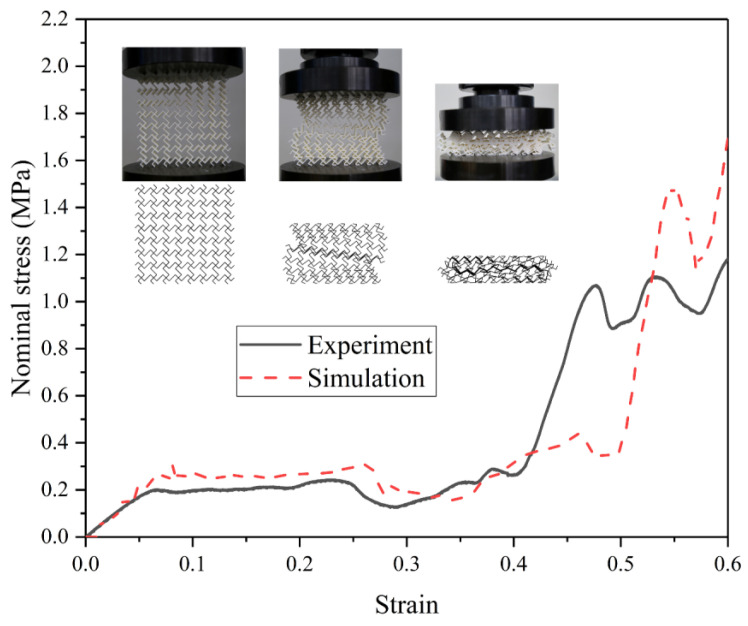
Nominal stress-strain curve of the quasi-static compression experiment and finite element model simulation.

The reliability of the simulation results depends critically on the accuracy of the finite element model. In this study, the constructed model is validated through quasi-static compression experiments. Tensile and honeycomb samples were fabricated using a fused deposition modeling (FDM) 3D printer (Raise3D Pro Plus, Raise3D, Shanghai, China) with PLA as the printing material. The dimensions of the tensile samples follow the specifications reported by Qi et al. [41]. The PLA material properties obtained from tensile testing are density ρ = 1.25 × 10^3^ kg/m^3^, Young’s modulus *E* = 2.75 GPa, yield stress $\sigma_{y}$ = 39.79 MPa, and Poisson’s ratio *v* = 0.35. Figure 4 presents a schematic of the TMRH-45-90 honeycomb sample and the quasi-static compression setup. An impact velocity of 2 mm/min was applied in the experiment. To reduce computational cost in Abaqus/Explicit, the verification simulation employed a higher constant velocity of 1 m/s. Figure 5 compares the nominal stress–strain curves obtained from experiments and simulations. The results show good agreement, particularly in the plateau region where stress values are nearly identical. Both the experimental and simulated samples enter the densification stage at a strain of approximately 0.4. Additionally, comparison of the deformation modes of TMRH-45-90 indicates that the finite element model reproduces the experimental deformation behavior accurately under equivalent strain. These observations confirm that the established finite element model possesses sufficient accuracy to support the analyses conducted in this study. It should be noted that the FE model employed for impact simulations adopts the same geometric configuration, mesh strategy, contact definitions, and boundary conditions as those used in the experimentally validated model, with the only difference being the constitutive material properties. Following established practices in the literature, the validated modeling framework is therefore considered applicable to impact analyses involving aluminum alloy materials.

## 4. Results and Discussion

This section is primarily based on a mechanics-based and deformation-mechanism-oriented framework to investigate the effects of key geometric parameters and loading conditions on symmetric honeycomb structures. Rather than deriving closed-form analytical solutions, the influence of impact velocity and geometric angles is elucidated through systematic comparisons of deformation modes, stress distributions, and energy absorption characteristics. These descriptors are widely recognized as effective theoretical tools for characterizing the mechanical behavior of honeycomb and architected metamaterial structures.

### 4.1. Comparison of Mechanical Properties of Different Honeycomb Structures

**Figure 6:**
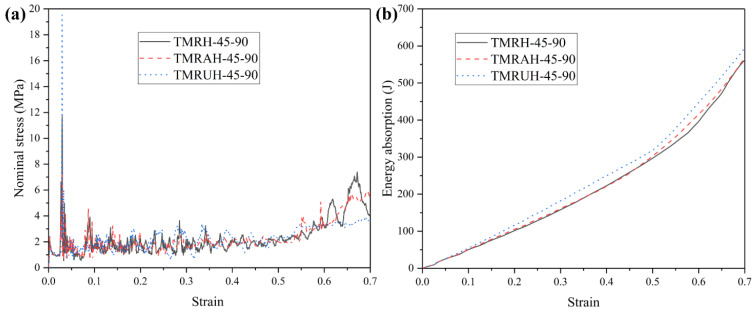
Nominal stress–strain and energy absorption behavior of tetra-missing rib honeycombs with different configurations: (**a**) Nominal stress–strain curves and (**b**) Energy absorption curves.

**Figure 7:**
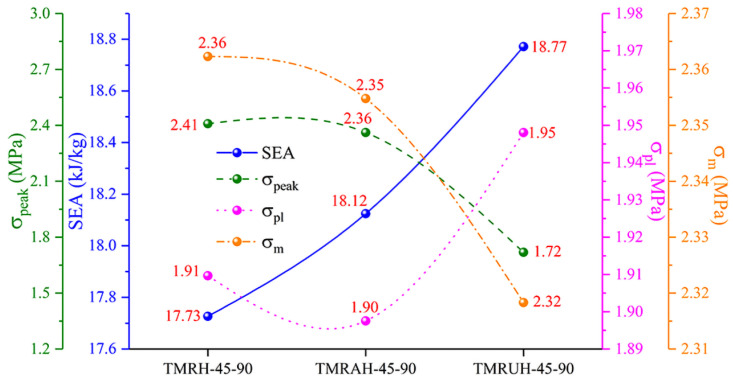
Mechanical property analysis of tetra-missing rib honeycombs with various configurations.

To investigate the two distinct symmetrical strategies represented by TMRAH-45-90 and TMRUH-45-90, as well as the mechanical behavior of TMRH-45-90, all three honeycomb configurations are analyzed. In this study, an impact velocity of 20 m/s is applied, and the wall thickness for all structures is maintained at 1.0 mm. The mass of the honeycomb structure is the same for the three different layout strategies analyzed in this section. Figure 6 shows the nominal stress-strain curves and energy absorption curves for different layout strategies and the same wall thickness. In Figure 6a, during the stage of platform stress, the nominal stresses of the honeycombs of the three configurations are almost the same, and the peak stress of TMRUH-45-90 is smaller than that of the honeycombs of the other two configurations, but its energy absorption is also better than that of the other two honeycomb configurations. Additionally, the energy absorption by TMRAH-45-90 is also greater than that of TMRH-45-90, and its peak stress is less than that of TMRH-45-90, which shows that two symmetrical arrangement strategies for tetra-missing rib honeycomb units can effectively improve the mechanical properties of conventional tetra-missing rib honeycomb. Figure 7 shows the results of the analysis of the mechanical performance of three honeycomb structures with the same wall thickness; the detailed data are shown in Table 1. It should be noted that, unless otherwise stated, all numerical results presented in the tables are obtained from deterministic finite element simulations based on the validated Abaqus/Explicit model. The experimental tests were conducted only for model validation purposes. Therefore, conventional measurement uncertainties associated with experimental data are not applicable to the majority of the tabulated results. As shown in Figure 7, the mechanical properties of TMRUH-45-90 are better than those of TMRH-45-90 and TMRAH-45-90, and the SEA values are 5.87% and 3.59% larger than those of TMRH-45-90 and TMRAH-45-90, respectively. The $\sigma_{peak}$ of TMRUH-45-90 is less than that of the two, which is more suitable for crashworthiness research, and the $\sigma_{pl}$ of TMRUH-45-90 is larger than that of the other two honeycombs. Although the mechanical properties of TMRAH-45-90 are not as good as those of TMRUH-45-90, its comprehensive mechanical properties are better than those of TMRH-45-90. Figure 7 shows that the SEA of TMRAH-45-90 itself is greater than that of TMRH-45-90, and its $\sigma_{peak}$ is less than that of TMRH-45-90. According to the above analysis, the introduction of symmetrical design can improve the mechanical properties of conventional tetra-missing rib honeycomb.

**Figure 8:**
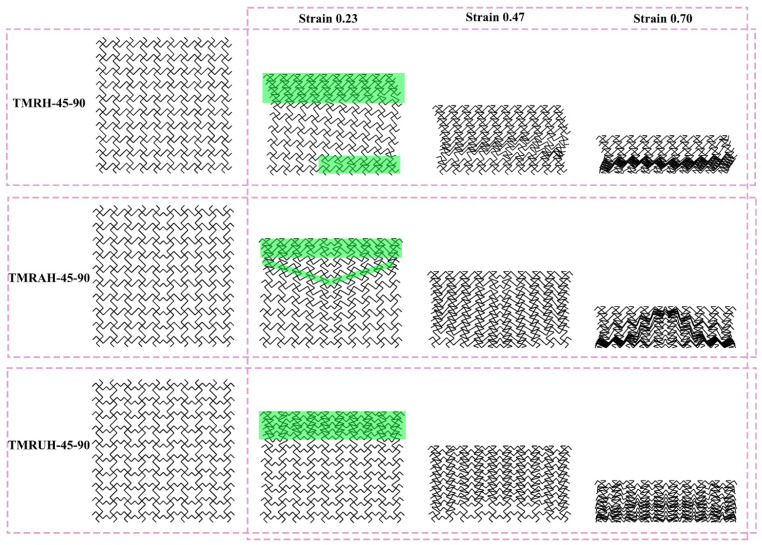
Deformation modes of TMRH-45-90, TMRAH-45-90, and TMRUH-45-90.

Figure 8 illustrates the deformation modes of TMRH-45-90, TMRAH-45-90, and TMRUH-45-90 under an impact velocity of 20 m/s. Comparison of the three honeycomb structures reveals that the initial deformation of TMRH-45-90 is primarily localized at the upper left and lower right corners, with local densification occurring during the mid-to-late stages of deformation. Due to their unique structural characteristics, TMRAH-45-90 and TMRUH-45-90 are more stable in deformation than TMRH-45-90. The initial deformation modes of TMRAH-45-90 are “I” type and “V” type, and the main characteristic of its force deformation is left and right symmetry. The initial deformation of TMRUH-45-90 occurs at the impact end; the deformation mode is mainly “I” type, and it gradually enters the densification stage with increasing strain. Based on the foregoing analysis, it can be concluded that the two symmetrical designs, TMRAH-45-90 and TMRUH-45-90, not only enhance the energy absorption of the tetra-missing rib honeycomb and reduce $\sigma_{peak}$, but also effectively mitigate the unstable deformation behavior of the structure.

### 4.2. Effect of Impact Velocity on Honeycomb Structure

**Figure 9:**
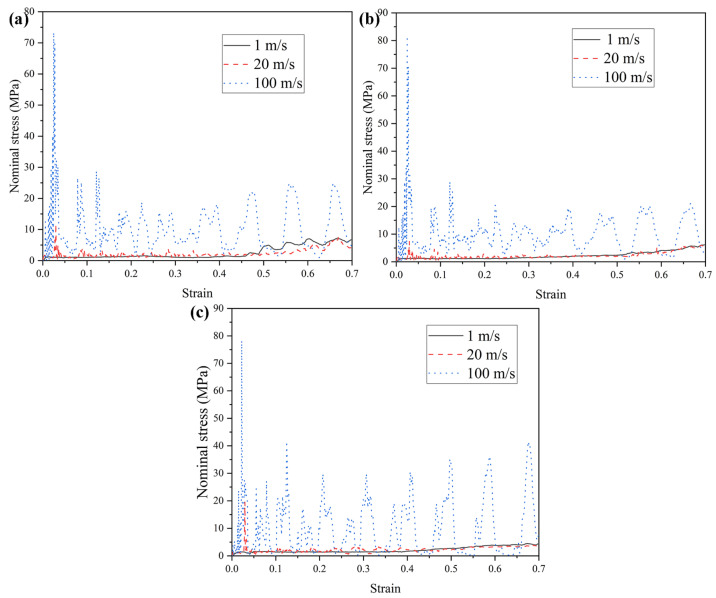
Nominal stress–strain curves of honeycomb structures at varying impact velocities: (**a**) TMRH-45-90, (**b**) TMRAH-45-90, and (**c**) TMRUH-45-90.

**Figure 10:**
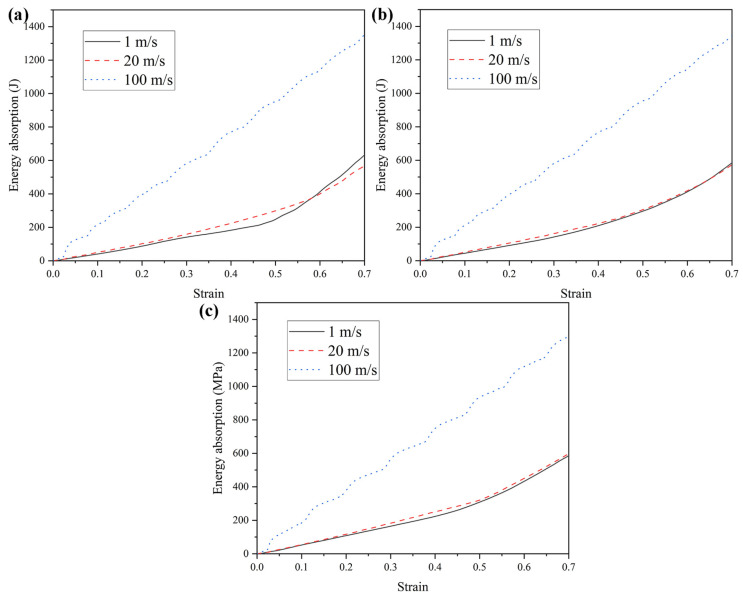
Energy absorption curves of honeycomb structures at varying impact velocities: (**a**) TMRH-45-90, (**b**) TMRAH-45-90, and (**c**) TMRUH-45-90.

Honeycomb structures are sensitive to impact velocity and typically exhibit varying mechanical responses and deformation modes under different loading rates. Accordingly, this section investigates the mechanical behavior and deformation patterns of three honeycomb configurations subjected to a range of impact velocities. The wall thickness is maintained at t = 1.0 mm, and three representative velocities are considered: low (1 m/s), medium (20 m/s), and high (100 m/s). It should be noted that the selected impact velocities are employed to investigate the dynamic deformation mechanisms and rate-dependent mechanical responses of the honeycomb structures, rather than to represent realistic vehicle impact scenarios. Such velocity levels are commonly adopted in numerical studies of cellular materials to reveal inertia effects and deformation mode transitions [74,75]. The mechanical performance of the honeycomb structures at these velocities is summarized in Table 2. Although TMRAH-45-90 and TMRH-45-90 exhibit very similar deformation modes under high-velocity impact, the $\sigma_{peak}$ mainly reflects the transient response at the initial loading stage, which is strongly influenced by geometric symmetry and inertia effects rather than the subsequent progressive collapse behavior. Figure 9 and Figure 10 present the corresponding nominal stress–strain curves and energy absorption profiles, respectively. The figure shows that different honeycomb structures have different mechanical properties corresponding to different impact velocities. For example, the energy absorption by TMRH-45-90 and TMRAH-45-90 under an impact of medium velocity is slightly less than for a low velocity impact, and only the energy absorption of TMRUH-45-90 improves with increasing impact velocity. However, the platform stresses of TMRH-45-90, TMRAH-45-90, and TMRUH-45-90 all show an increase in oscillation amplitude with increasing impact velocity, which is due to the different types of deformation of the honeycomb structure at different impact velocities, resulting in such changes in the nominal stress-strain curves of the honeycomb structures. The significantly higher energy absorption observed at an impact velocity of 100 m/s can be attributed to the combined effects of inertia confinement and strain-rate sensitivity. At high-impact velocities, inertial effects suppress premature local buckling and promote a more progressive and stable crushing mode, resulting in increased plastic deformation and higher energy dissipation. In contrast, the responses at 1 m/s and 20 m/s remain governed by similar deformation mechanisms, leading to comparable energy absorption levels.

**Figure 11:**
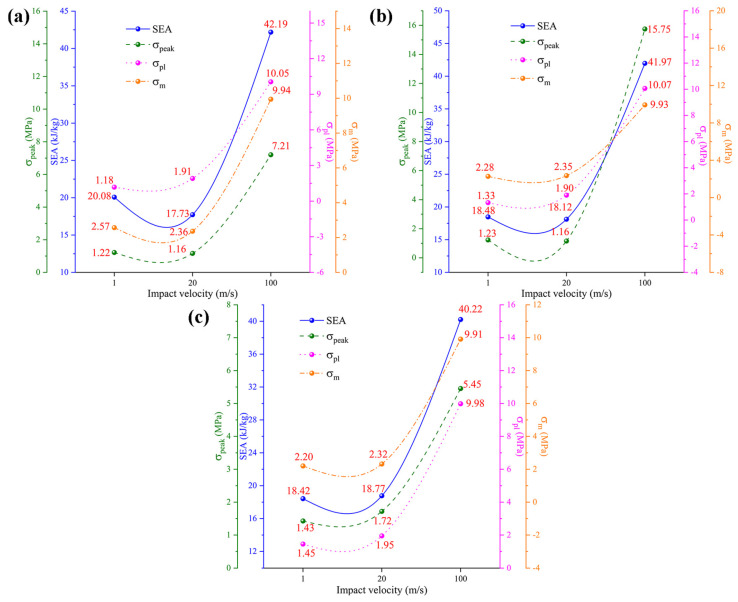
Analysis of the results for the mechanical properties of TMRH, TMRAH, and TMRUH under different impact velocities: (**a**) TMRH-45-90, (**b**) TMRAH-45-90, and (**c**) TMRUH-45-90.

The analysis results of the mechanical properties of the honeycomb structures at different impact velocities are presented in Figure 11. The data in Figure 11 show that, under the three impact velocities, TMRH-45-90 and TMRAH-45-90, except for ${\;\sigma }_{m}$, SEA, $\sigma_{peak},$ and $\sigma_{pl}$, improve to varying degrees with increasing impact velocity. For example, under high-velocity impact, the SEA of TMRH-45-90 increases by 110.11% and 137.96% compared with low- and medium-velocity impacts, respectively, and $\sigma_{pl}$ increases by 751.69% and 426.18% compared with the other two impact velocities. Compared with low-velocity and medium-velocity impacts, the SEA of TMRAH-45-90 under high-velocity impact increases by 127.11% and 131.62%, respectively. Compared with TMRH-45-90 and TMRAH-45-90, the mechanical properties of TMRUH-45-90 improve with increasing impact velocity. Among them, the SEA of TMRUH-45-90 under the high-velocity impact is less, and that of the medium-velocity impact is increased by 118.35%, and114.28%, respectively, and $\sigma_{pl}$ increases by 588.28% and 411.79% compared with the other two velocities. This can also give preliminary evidence that increasing the impact velocity has a positive effect on the mechanical properties of TMRUH-45-90. This trend provides preliminary evidence that increasing impact velocity can activate inertia-dominated deformation mechanisms, which enhances the energy dissipation efficiency of TMRUH-45-90 under dynamic loading conditions. From a practical perspective, the observed enhancement of specific energy absorption (SEA) at high impact velocities indicates that the proposed honeycomb structures are particularly suitable for dynamic protection scenarios rather than quasi-static load-bearing applications. In high-velocity events, such as vehicle collisions, debris impact, or blast-induced loading, structural components are required to dissipate kinetic energy efficiently within a limited deformation distance. The increased SEA at high velocities suggests that honeycomb architectures can convert a larger portion of the input kinetic energy into plastic dissipation through progressive crushing, thereby improving impact-mitigation capability. This velocity-sensitive energy absorption behavior makes the proposed structures promising candidates for lightweight protective systems in transportation, aerospace shielding, and crashworthy components. Moreover, the relatively stable energy absorption at low and medium velocities implies that the structures maintain predictable mechanical responses across a wide range of loading rates, which is advantageous for engineering design under uncertain impact conditions.

**Figure 12:**
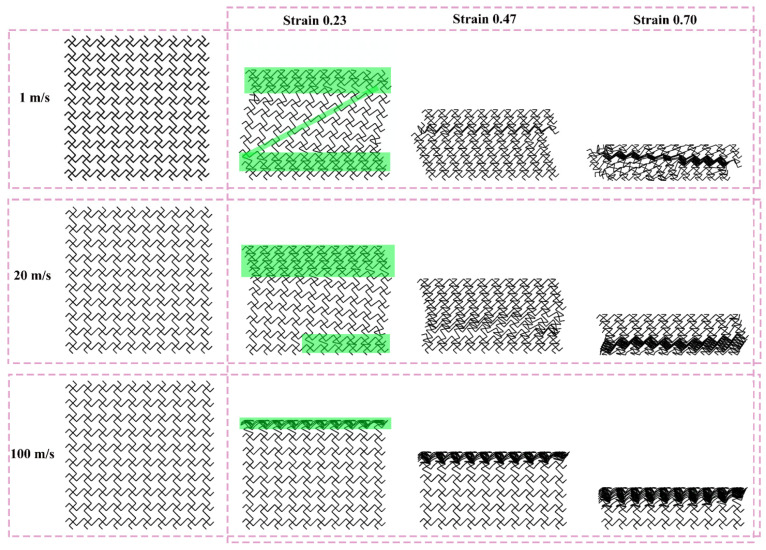
Deformation modes of TMRH-45-90 under different impact velocities.

**Figure 13:**
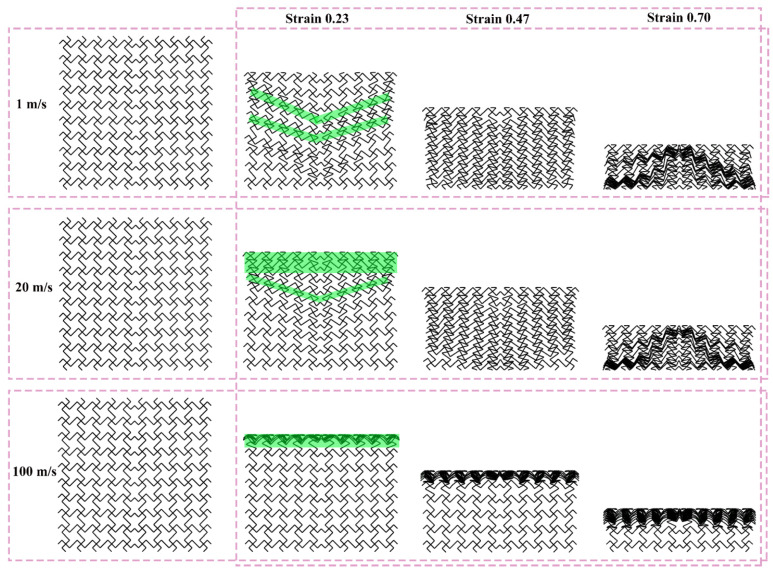
Deformation modes of TMRAH-45-90 under different impact velocities.

**Figure 14:**
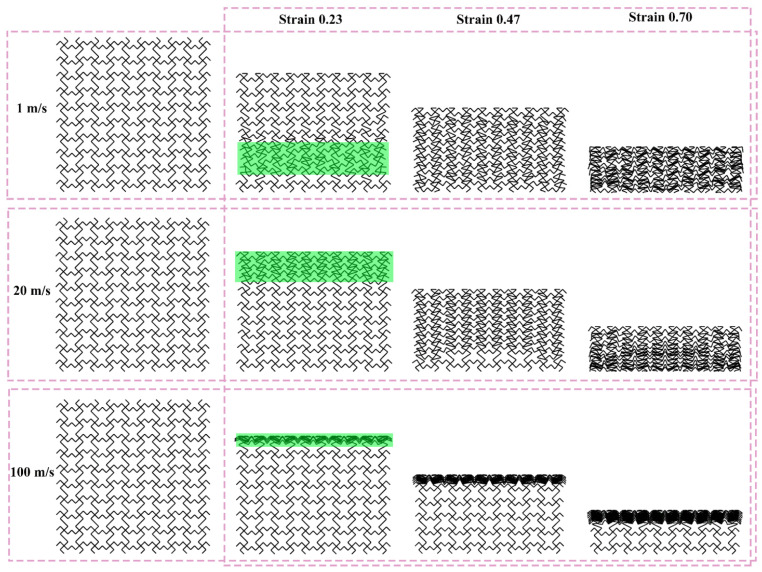
Deformation modes of TMRUH-45-90 under different impact velocities.

Figure 12, Figure 13, and Figure 14 show the deformation modes of TMRH-45-90, TMRAH-45-90, and TMRUH-45-90 at different impact velocities, respectively. In Figure 12, the deformation modes of TMRH-45-90 have different characteristics at different speeds. Taking 1 m/s as an example, TMRH-45-90 is deformed into a Z-shape at the initial stage, and then locally densified, and finally it gradually enters densified deformation. At an impact velocity of 20 m/s, the initial deformation of TMRH-45-90 also has a “Z-shape deformation, but then a preliminary densification deformation occurs locally near the fixed end. At an impact velocity of 100 m/s, TMRH-45-90 collapses from top to bottom in an I-shape deformation because the inertia effect increases with increasing impact velocity. It then undergoes deformation into an I-shape and enters the compaction stage. In Figure 13, TMRAH-45-90 has a special deformation mode due to its unique arrangement and layout. At impact velocities of 1 m/s and 20 m/s, TMRAH-45-90 exhibits a pronounced negative Poisson’s ratio behavior during the initial deformation stage, with an overall V-shaped deformation mode. Beyond a strain of 0.47, the structure gradually enters the densification stage. Under a high impact velocity of 100 m/s, the inertial effects become more significant, weakening the negative Poisson’s ratio effect and leading to an I-shaped deformation mode. Figure 14 shows the deformation mode of TMRUH-45-90 at different velocities. Because of the unique symmetrical arrangement strategy of TMRUH-45-90, its deformation does not change significantly due to the change in impact velocity, and it can be considered an I-shape deformation. The difference is that the initial deformation position and the number of deformations are different. At an impact velocity of 1 m/s, the deformation of TMRUH-45-90 occurs first at the bottom, then it gradually evolves into overall deformation until it enters the densification stage at the end of deformation. Under medium-velocity and high-speed impact, the initial deformation of TMRUH-45-90 occurs at the impact end, and the difference is due to the influence of the inertial effect. The number of deformed elements of TMRUH-45-90 is significantly larger at medium impact velocity than at high impact velocity.

### 4.3. Effect of Angle on Mechanical Properties of Honeycomb

#### 4.3.1. The Effect of θ on the Mechanical Properties of Honeycomb

**Figure 15:**
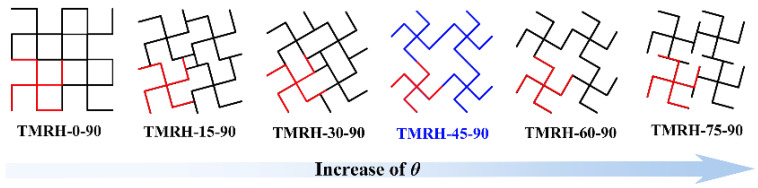
Evolution process of honeycomb structure subunits of TMRH with different angles *θ*.

**Figure 16:**
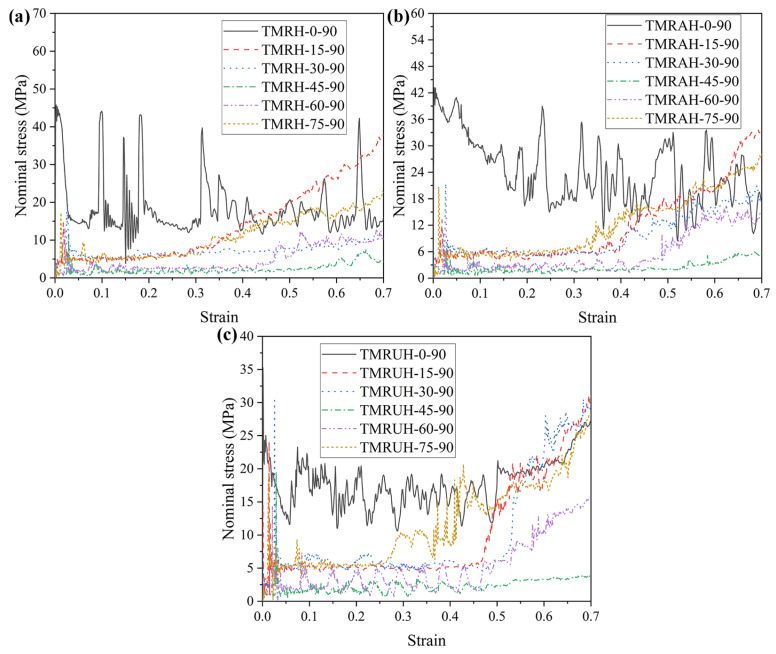
Nominal stress-strain curves of structures with different *θ* at an impact velocity of 20 m/s: (**a**) TMRH, (**b**) TMRAH, and (**c**) TMRUH.

**Figure 17:**
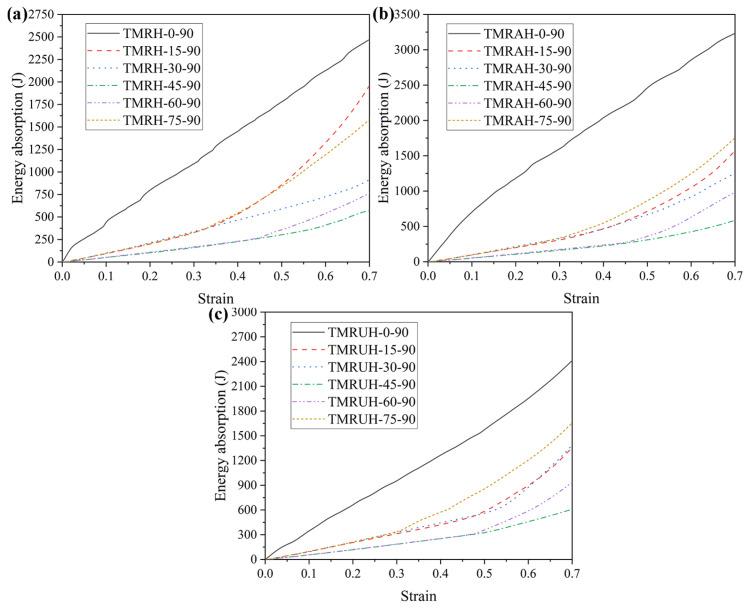
Energy absorption curves of honeycomb structures with different *θ* at an impact velocity of 20 m/s: (**a**) TMRH, (**b**) TMRAH, and (**c**) TMRUH.

The tetra-missing rib honeycomb is a special form when the *r* (cylinder radius) of the tetra-chiral honeycomb is 0. Based on the characteristics of the tetra-chiral honeycomb [41], the mechanical properties and deformation modes of the two angles (*θ* and *φ*) in Figure 2 are studied at different angles in this section. To explore the influence of different *θ* on the mechanical properties of the honeycomb structure, this section studies the mechanical properties of the honeycomb for six angles: *θ* = 0, *θ* = 15, *θ* = 30, *θ* = 45, *θ* = 60, and *θ* = 75. Figure 15 shows the evolution of the tetra-missing rib honeycomb unit at different *θ*. In this process, *φ* = 90 remains unchanged, the wall thickness remains 1.0 mm, and the impact velocity is 20 m/s. Figure 16 and Figure 17 present the nominal stress–strain curves and energy absorption curves, respectively, for structures with varying θ values. Under different *θ*, the nominal stresses of TMRH, TMRAH, and TMRUH have almost the same variation. Among them, the leading advantage of the nominal stress curve at *θ* = 0 is the most obvious, and its energy absorption is also the largest, but the fluctuation range of the curve is also the largest because the tetra-missing rib honeycomb array at this angle can be approximately regarded as a quadrilateral honeycomb. At this angle, the vertical ribs in the honeycomb structure undergo plastic deformation, which absorbs energy well. When other honeycomb structures under *θ* are deformed, almost all of their subunits undergo torsional deformation of plastic hinges. Therefore, when *θ* = 45, the horizontal component force of the tetra-missing rib honeycomb unit is the largest, which leads to the smallest nominal stress and the smallest energy absorption at this angle. When *θ* > 45, the nominal stresses of TMRH, TMRAH, and TMRUH gradually increase, and their energy absorption improves to a certain extent. These results provide preliminary evidence of how the nominal stress and energy absorption of TMRH, TMRAH, and TMRUH vary with different *θ* values. As *θ* increases, both the nominal stress and energy absorption of the three honeycomb configurations initially decrease and subsequently increase.

**Figure 18:**
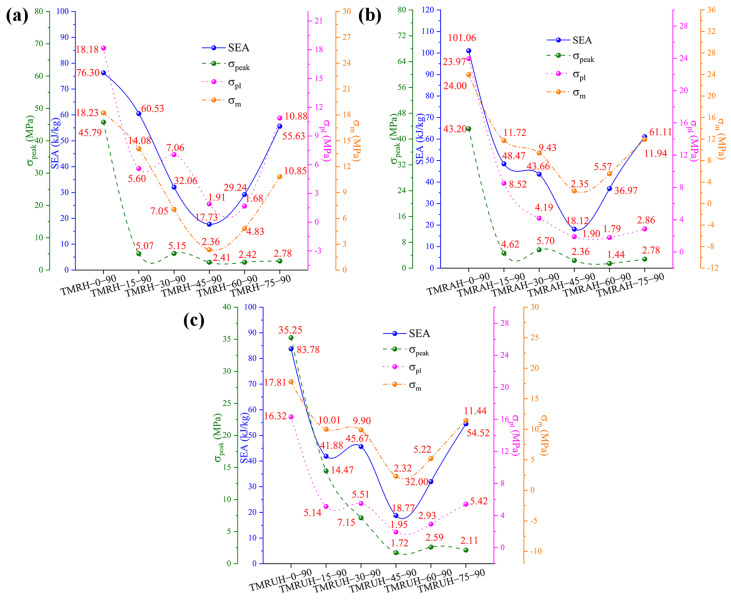
Mechanical properties of TMRH, TMRAH, and TMRUH with different *θ* at an impact velocity of 20 m/s: (**a**) TMRH, (**b**) TMRAH, and (**c**) TMRUH.

The mechanical behavior of the honeycomb structures at different *θ* values is presented in Figure 18, with detailed data summarized in Table 3. Figure 18a specifically illustrates the mechanical properties of TMRH as a function of *θ*. With increasing *θ*, the SEA and $\sigma_{m}$ of TMRH first decrease and then increases, which confirms the behavior we found through Figure 16 and Figure 17. When *θ* = 0, the honeycomb structure is more special, which is not discussed here. It is obvious that the SEA and $\sigma_{m}$ of TMRH-15-90 are the most advanced; compared with TMRH-45-90, they increase by 330.34% and 672.46%, respectively. Second, the mechanical properties of TMRH-75-90 are also more advanced. Its $\sigma_{peak}$ is only 2.78 MPa, but its SEA and $\sigma_{m}$ are 3.14 times and 4.61 times those of TMRH-45-90, respectively. Figure 18b and Figure 18c show the mechanical properties of TMRAH and TMRUH, respectively. The data in the figure show that the mechanical properties of TMRAH and TMRUH maintain almost the same variation as TMRH. From the data in the figure, the mechanical properties of TMRAH and TMRUH maintain almost the same variation as TMRH, but because of their own structural characteristics under different *θ* conditions, the various mechanical properties maintain a leading position to varying degrees. Taking *θ* = 15 and *θ* = 30 as examples, when *θ* = 15, the SEA of TMRAH-15-90 is 48.47 kJ/kg, which is 15.74% more than TMRUH-15-90. When *θ* = 30, the SEA of TMRUH-30-90 is 45.67 kJ/kg, which is 4.60% larger than that of TMRAH-30-90.

**Figure 19:**
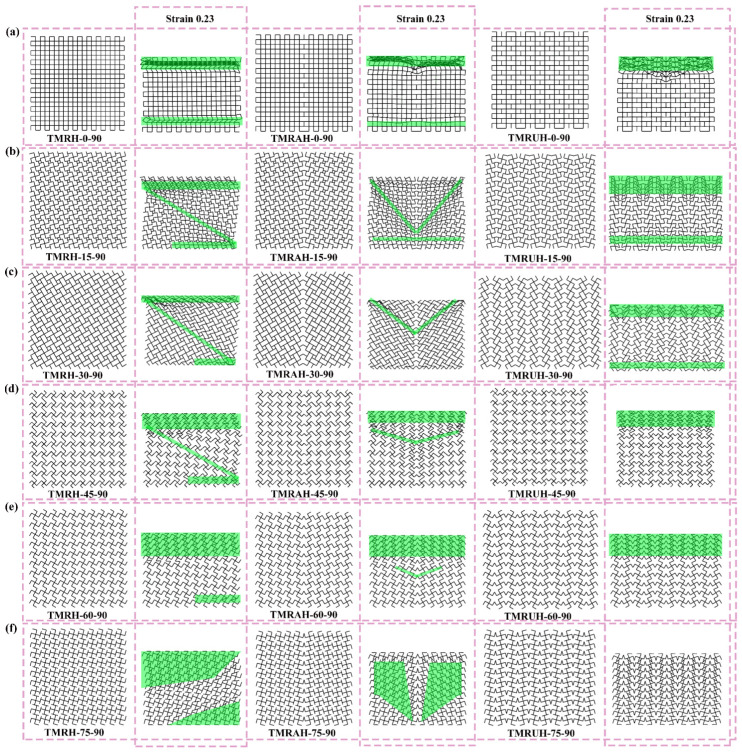
Deformation modes of TMRH, TMRAH, and TMRUH with different *θ* at an impact velocity of 20 m/s: (**a**) *θ* = 0, (**b**) *θ* = 15, (**c**) *θ* = 30, (**d**) *θ* = 45, (**e**) *θ* = 60, and (**f**) *θ* = 75.

Figure 19 illustrates the deformation modes of TMRH, TMRAH, and TMRUH at different *θ* values. The figure specifically depicts the deformation patterns of the three structures during the initial deformation stage (strain = 0.23). The deformation of TMRH, TMRAH, and TMRUH has different deformations due to their own structural characteristics, and the deformation mode when *θ* = 0 is the most special because at this angle, TMRH, TMRAH, and TMRUH can be approximately regarded as quadrilateral honeycombs. The deformation mode is also shown as an I-shape; the difference is that when TMRH and TMRAH are impacted, both the top and bottom of the honeycomb are deformed, while when TMRUH is impacted, only the top of the honeycomb is deformed. Under different *θ*, TMRH exhibits different degrees of Z-shape deformation. As *θ* increases, the initial deformation of TMRH gradually evolves to occur in the upper left and lower right corners of the honeycomb. The deformation of TMRAH has obvious characteristics. Its local deformation modes are mainly V-shape and I-shape. As the angle *θ* increases, the deformation mode of the TMRAH gradually evolves into overall deformation, the force deformation of the TMRAH is obviously more stable, and its deformation has different degrees of a negative Poisson’s ratio effect. The deformation mode of TMRUH is mainly I-shape, and as the angle *θ* increases, its deformation gradually evolves from local to overall collapse.

#### 4.3.2. Effect of φ on the Mechanical Properties of Honeycomb

**Figure 20:**
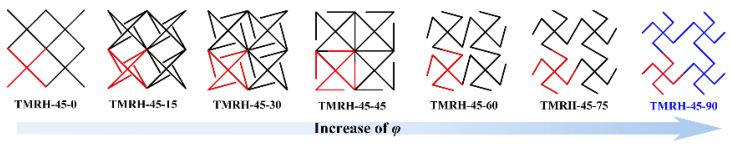
Evolution of the honeycomb structure unit of TMRH with different *φ*.

**Figure 21:**
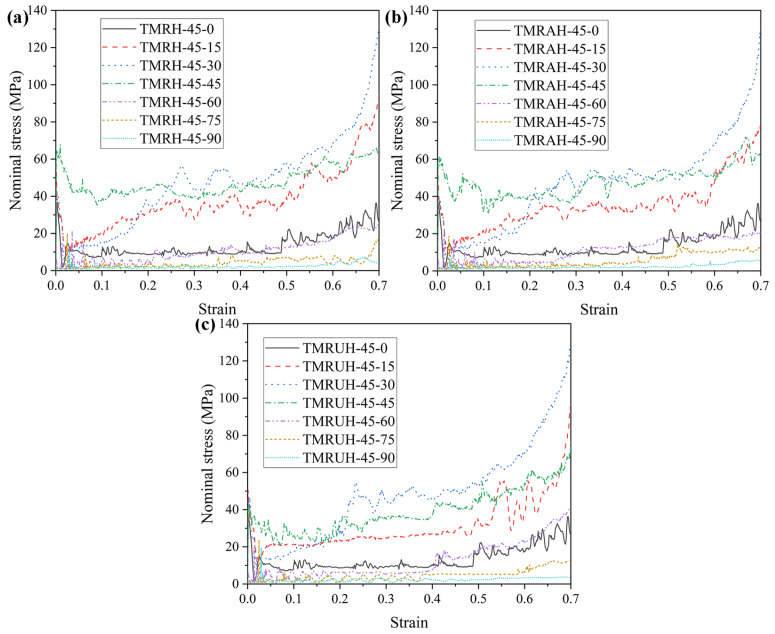
Nominal stress-strain curves of TMRH, TMRAH and TMRUH with different *φ* at an impact velocity of 20 m/s: (**a**) TMRH, (**b**) TMRAH and (**c**) TMRUH.

**Figure 22:**
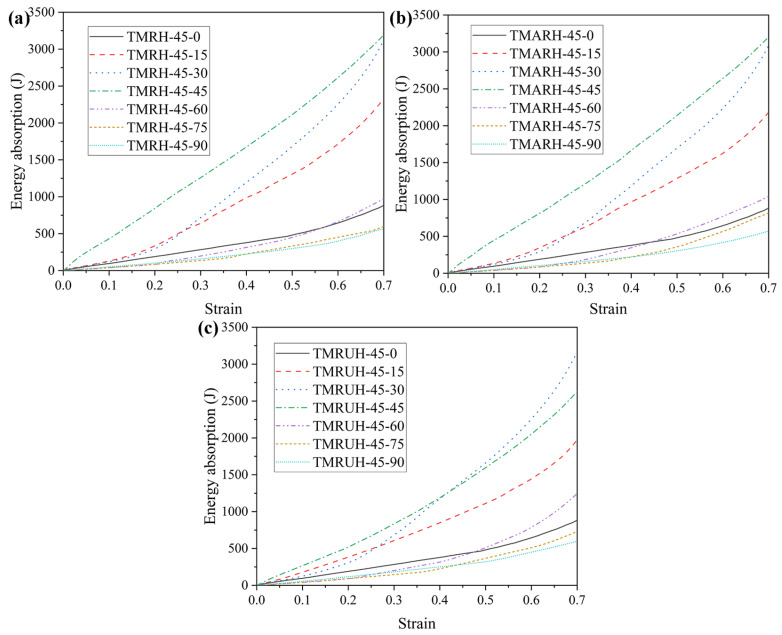
Energy absorption curves of TMRH, TMRAH, and TMRUH with different *φ* at an impact velocity of 20 m/s: (**a**) TMRH, (**b**) TMRAH, and (**c**) TMRUH.

To explore the influence of different *φ* values on the mechanical properties of the honeycomb structure, this section analyzes the honeycomb structure for the seven angles *φ* = 0, *φ* = 15, *φ* = 30, φ = 45, *φ* = 60, *φ* = 75, and *φ* = 90. The research on mechanical properties in Figure 20 shows the evolution of the tetra-missing rib honeycomb unit at different *φ*. In this process, *θ* = 90 remains unchanged, the wall thickness is *t* = 1.0 mm, and the impact velocity is 20 m/s. Figure 21 and Figure 22 present the nominal stress–strain curves and energy absorption curves, respectively, for TMRH, TMRAH, and TMRUH at different *φ* values. The figure shows that the nominal stresses of TMRH, TMRAH, and TMRUH undergo different changes with changing *φ*. When the strain is 0.7, TMRH-45-30, TMRAH-45-30, and TMRUH-45-30 enter the densification stage, and the nominal stress and energy absorption are almost the best among the seven angles for each type, while TMRH-45-90, TMRAH-45-90, and TMRUH-45-90 have the lowest nominal stress and energy absorption. By observing the energy absorption curves of TMRH, TMRAH, and TMRUH, it is clear that when *φ* = 15, 30, and 45, the energy absorption by TMRH, TMRAH and TMRUH is advantageous compared with the honeycombs with other *φ* values.

**Figure 23:**
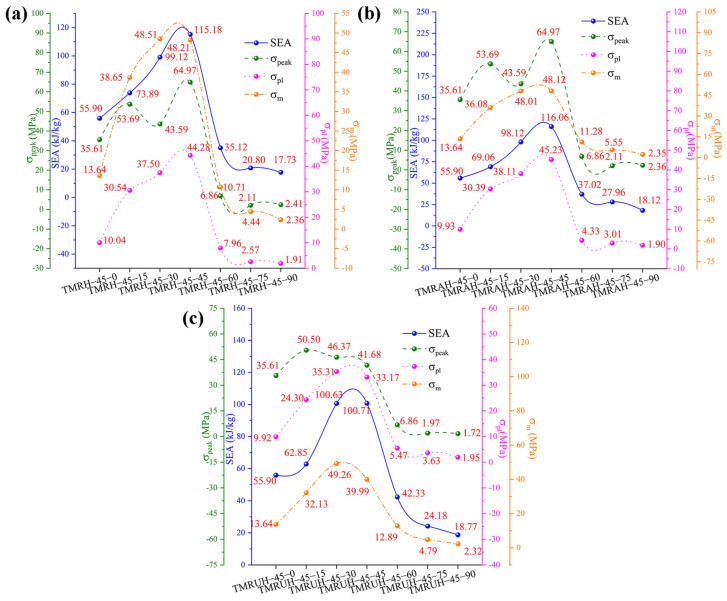
Mechanical properties of TMRH, TMRAH, and TMRUH with different *φ* at an impact velocity of 20 m/s: (**a**) TMRH, (**b**) TMRAH, and (**c**) TMRUH.

Figure 23 shows the mechanical properties of TMRH, TMRAH, and TMRUH at different *φ* values, and the detailed mechanical property data are shown in Table 4. Figure 23 shows that the changes in SEA, $\sigma_{peak}$, $\sigma_{pl}$ and $\sigma_{m}$ with *φ* are almost exactly opposite to that of *θ*. For different *φ*, the changes in the mechanical properties of TMRH, TMRAH, and TMRUH are increased first, then decreased. The mechanical properties of TMRH are shown in Figure 23a. The SEA, $\sigma_{peak}$, $\sigma_{pl}$ and $\sigma_{m}$ of TMRH-45-45 are the best compared with other TMRHs at the same *φ*. At these seven angles, the mechanical properties of TMRH-45-90 are relatively small. A comparison shows that, compared with TMRH-45-90, the SEA, $\sigma_{pl}$ and $\sigma_{m}$ of TMRH-45-45 increase by 549.63%, 2218.32% and 1942.80%, respectively. The mechanical properties of TMRAH and TMRUH are shown in Figure 23b and Figure 23c, respectively. As two different symmetrically arranged honeycombs, their mechanical properties are slightly different. For example, the SEA, $\sigma_{peak}$, $\sigma_{pl}$ and $\sigma_{m}$ of TMRAH-45-45 all maintain the best values in the same type of honeycomb, and its SEA, $\sigma_{pl}$ and $\sigma_{m}$ are 540.51%, 2280.53%, and 1947.66% better than those of TMRAH-45-90, which has the smallest mechanical properties, respectively. When *φ* = 30, the $\sigma_{peak}$ of TMRAH-45-30 is less than that of TMRAH-45-15. For TMRUH-45-45, only the SEA is the best, which is 436.55% ahead of TMRUH-45-90, while $\sigma_{pl}$ and $\sigma_{m}$ in the mechanical properties of TMRUH-45-30 are the highest among the same type of honeycombs, which are 1710.77% and 2023.28% greater than those of TMRUH-45-90, respectively.

**Figure 24:**
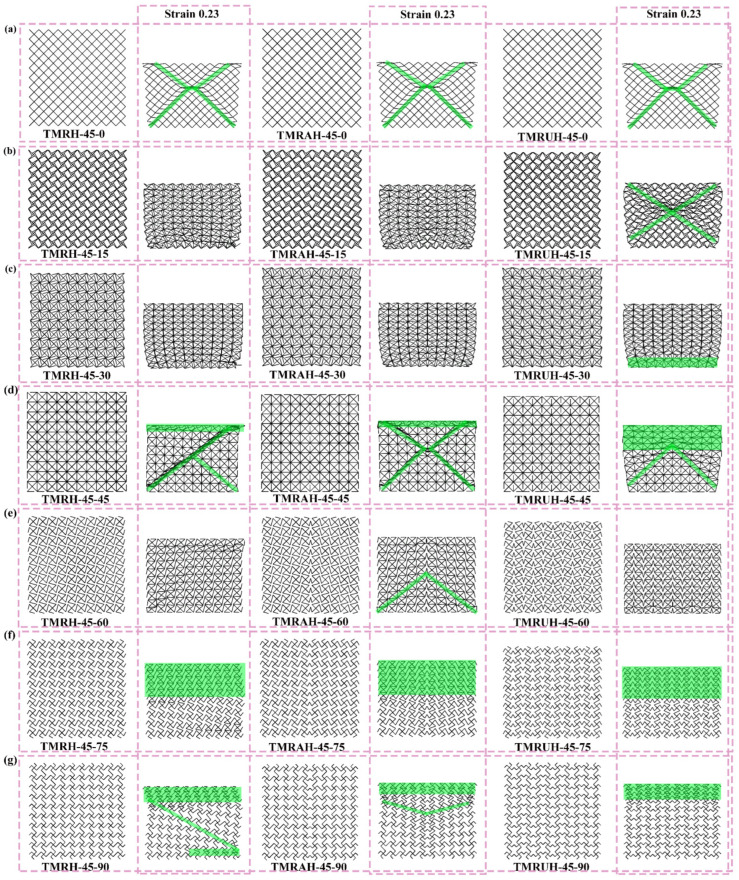
Deformation modes of TMRH, TMRAH, and TMRUH with different *φ* at an impact velocity of 20 m/s: (**a**) *φ* = 0, (**b**) *φ* = 15, (**c**) *φ* = 30, (**d**) *φ* = 45, (**e**) *φ* = 60, (**f**) *φ* = 75, and (**g**) *φ* = 90.

Figure 24 shows the deformation modes of TMRH, TMRAH, and TMRUH at different *φ*. When *φ* = 0, TMRH, TMRAH, and TMRUH can all be regarded as diamond-shaped honeycombs, and thus all their deformation modes are X-shape. The initial deformation mode of TMRH-45-90 is Z-shape, and the deformation modes of other TMRHs can be regarded as the same type of deformation mode. The common feature of their deformation is that after the honeycomb subunit is impacted, the plastic hinge undergoes rotational deformation. For TMRAH, the deformation modes produced by TMRH-45-45 are X-shape and I-shape, and the deformation mode of TMRH-45-60 is V-shape. Similar to TMRH-45-75, the deformation mode of TMRAH-45-75 is initially manifested as plastic deformation at the impact end, TMRAH-45-90 exhibits I- and V-shape deformation modes, and the initial deformation of the other TMRAHs for several φ values is the rotational deformation of the plastic hinge of the honeycomb subunit. The stress deformation of TMRUH is relatively simple. The deformation mode of TMRUH-45-15 is X-shape, while the initial deformation of TMRUH-45-45 is I-shape and V-shape. Except for TMRUH-45-60, which is the torsional deformation of the subunit, the other angles all show I-shape deformation modes. The difference is that the deformation of TMRUH-45-30 is at the bottom of the honeycomb structure, while TMRUH-45-75 and TMRUH-45-90 deform at the impacted end of the honeycomb.

## 5. Conclusions

In this study, two symmetric-strategy honeycomb structures, TMRAH and TMRUH, were proposed based on an analysis of the deformation modes of tetra-missing rib honeycombs. A finite element model of the honeycomb was developed using Abaqus/Explicit and validated through comparison with quasi-static experiments. The mechanical properties and deformation behaviors of the tetra-missing rib honeycombs, TMRAH, and, TMRUH were systematically compared. Further investigations were conducted to evaluate their mechanical performance and deformation modes under varying impact velocities and geometric angles. The scope of the present work is intentionally limited to the investigation of impact-induced mechanical behavior and deformation mechanisms of symmetric honeycomb architectures, which are critical for crashworthiness-related applications. Experimental and numerical studies under additional loading conditions, including bending and fatigue, will be systematically addressed in future work.

In addition, the advantages of the investigated materials are clarified. Aluminum alloy, employed in the numerical simulations, provides high specific strength, excellent energy absorption capability, and good recyclability, making it suitable for engineering-oriented crashworthiness applications. PLA was adopted in the experimental validation due to its compatibility with additive manufacturing, which enables efficient fabrication of complex symmetric honeycomb architectures and rapid verification of deformation mechanisms under compression-dominated loading. Although PLA exhibits relatively low tensile strength, it is adequate for the present compressive tests and does not affect the generality of the structural conclusions. The principal findings of this study can be summarized as follows:

(1) The mechanical behavior and deformation patterns of TMRH-45-90, TMRAH-45-90, and TMRUH-45-90 with identical wall thickness were analyzed. The results demonstrate that introducing symmetric layouts promotes more uniform and stable deformation compared with the conventional tetra-missing rib honeycomb, while maintaining comparable energy absorption capability. In particular, the TMRUH configuration exhibits an enhanced negative Poisson’s ratio (NPR) effect and improved mechanical performance. In contrast, TMRAH shows mechanical properties comparable to those of the original TMRH within numerical accuracy, but benefits from improved structural symmetry, leading to more uniform deformation and suppression of asymmetric local collapse.
(2) The mechanical behavior and deformation modes of the three tetra-missing rib honeycomb types were evaluated under different impact velocities. The results demonstrate that the structures exhibit velocity-dependent mechanical responses. Specifically, the mechanical performance of TMRUH-45-90 increases with impact velocity, whereas TMRH-45-90 and TMRAH-45-90 show a slight reduction in mechanical properties at medium impact velocity.
(3) The influence of *θ* on the three honeycomb structures was analyzed. The honeycomb structures corresponding to different *θ* values had different structural characteristics and exhibited different plastic deformation modes and mechanical properties for medium-velocity impacts. According to the analysis of the simulation results, the nominal stress and energy absorption of TMRH, TMRAH, and TMRUH varied under different *θ*. As *θ* increased, the nominal stress and energy absorption of TMRH, TMRAH, and TMRUH decreased first, after increasing.
(4) The effect of *φ* on the mechanical properties of TMRH, TMRAH, and TMRUH was determined. The research results showed that when *φ* = 15, 30, and 45, energy absorption by TMRH, TMRAH, and TMRUH had a clear advantage compared with honeycombs with other φ values. The mechanical properties of the three honeycomb structures first increased and then decreased with increasing *φ*.

## Figures and Tables

**Table 1:** Results of the analysis of the mechanical properties of tetra-missing rib honeycomb with different configurations.

Honeycomb	Thickness (mm)	Mass (kg)	EA (J)	SEA (kJ/kg)	$\sigma_{peak}$ (MPa)	$\sigma_{pl}$ (MPa)	$\sigma_{m}$ (MPa)
TMRH-45-90	1.00	0.0324	574.35	17.73	2.41	1.91	2.36
TMRAH-45-90	1.00	0.0324	587.24	18.12	2.36	1.90	2.35
TMRUH-45-90	1.00	0.0324	608.21	18.77	1.72	1.95	2.32

**Table 2:** Analysis of the results of the mechanical properties of tetra-missing rib honeycomb with different configurations under different impact velocities.

Honeycomb	Velocity (m/s)	Mass (kg)	Thickness (mm)	EA (J)	SEA (kJ/kg)	$\sigma_{peak}$ (MPa)	$\sigma_{pl}$ (MPa)	$\sigma_{m}$ (MPa)
TMRH-45-90	1	0.0324	1.00	650.44	20.08	1.22	1.18	2.57
TMRH-45-90	20	0.0324	1.00	574.35	17.73	1.16	1.91	2.36
TMRH-45-90	100	0.0324	1.00	1366.82	42.19	7.21	10.05	9.94
TMRAH-45-90	1	0.0324	1.00	598.86	18.48	1.23	1.33	2.28
TMRAH-45-90	20	0.0324	1.00	587.2373	18.12	1.16	1.90	2.35
TMRAH-45-90	100	0.0324	1.00	1359.783	41.97	15.75	10.07	9.93
TMRUH-45-90	1	0.0324	1.00	596.85	18.42	1.43	1.45	2.20
TMRUH-45-90	20	0.0324	1.00	608.21	18.77	1.72	1.95	2.32
TMRUH-45-90	100	0.0324	1.00	1303.00	40.22	5.45	9.98	9.91

**Table 3:** Mechanical properties of TMRH, TMRAH, and TMRUH with different *θ* at an impact velocity of 20 m/s.

Honeycomb	Mass (kg)	Thickness (mm)	θ (°)	φ (°)	EA (J)	SEA (kJ/kg)	$\sigma_{peak}$ (MPa)	$\sigma_{pl}$ (MPa)	$\sigma_{m}$ (MPa)
TMRH-0-90	0.0324	1.00	0	90	2472.00	76.30	45.79	18.18	18.23
TMRH-15-90	0.0324	1.00	15	90	1961.30	60.53	5.07	5.60	14.08
TMRH-30-90	0.0285	1.00	30	90	913.64	32.06	5.15	7.06	7.05
TMRH-45-90	0.0324	1.00	45	90	574.35	17.73	2.41	1.91	2.36
TMRH-60-90	0.0262	1.00	60	90	765.97	29.24	2.42	1.68	4.83
TMRH-75-90	0.0285	1.00	75	90	1585.46	55.63	2.78	10.88	10.85
TMRAH-0-90	0.032	1.00	0	90	3234.04	101.06	43.20	23.97	24.00
TMRAH-15-90	0.0324	1.00	15	90	1570.58	48.47	4.62	8.52	11.72
TMRAH-30-90	0.0287	1.00	30	90	1253.03	43.66	5.70	4.19	9.43
TMRAH-45-90	0.0324	1.00	45	90	587.24	18.12	2.36	1.90	2.35
TMRAH-60-90	0.0266	1.00	60	90	983.44	36.97	1.44	1.79	5.57
TMRAH-75-90	0.0287	1.00	75	90	1753.97	61.11	2.78	2.86	11.94
TMRUH-0-90	0.0288	1.00	0	90	2412.95	83.78	35.25	16.32	17.81
TMRUH-15-90	0.0324	1.00	15	90	1356.77	41.88	14.47	5.14	10.01
TMRUH-30-90	0.0304	1.00	30	90	1388.33	45.67	7.15	5.51	9.90
TMRUH-45-90	0.0324	1.00	45	90	608.21	18.77	1.72	1.95	2.32
TMRUH-60-90	0.0293	1.00	60	90	937.55	32.00	2.59	2.93	5.22
TMRUH-75-90	0.0304	1.00	75	90	1657.47	54.52	2.11	5.42	11.44

**Table 4:** Analysis of the results of the mechanical properties of TMRH, TMRAH, and TMRUH with different *φ* at an impact velocity of 20 m/s.

Honeycomb	Mass (kg)	Thickness (mm)	θ (°)	φ (°)	EA (J)	SEA (kJ/kg)	$\sigma_{peak}$ (MPa)	$\sigma_{pl}$ (MPa)	$\sigma_{m}$ (MPa)
TMRH-45-0	0.0162	1.00	45	0	905.56	55.90	35.61	10.04	13.64
TMRH-45-15	0.0324	1.00	45	15	2394.03	73.89	53.69	30.54	38.65
TMRH-45-30	0.0324	1.00	45	30	3211.38	99.12	43.59	37.50	48.51
TMRH-45-45	0.0281	1.00	45	45	3236.53	115.18	64.97	44.28	48.21
TMRH-45-60	0.0285	1.00	45	60	1000.96	35.12	6.86	7.96	10.71
TMRH-45-75	0.0297	1.00	45	75	617.89	20.80	2.11	2.57	4.44
TMRH-45-90	0.0324	1.00	45	90	574.35	18.77	1.72	1.95	2.32
TMRAH-45-0	0.0162	1.00	45	0	905.56	55.90	35.61	9.93	13.64
TMRAH-45-15	0.0324	1.00	45	15	2237.59	69.06	53.69	30.39	36.08
TMRAH-45-30	0.0324	1.00	45	30	3179.09	98.12	43.59	38.11	48.01
TMRAH-45-45	0.028	1.00	45	45	3249.78	116.06	64.97	45.23	48.12
TMRAH-45-60	0.0287	1.00	45	60	1062.54	37.02	6.86	4.33	11.28
TMRAH-45-75	0.0299	1.00	45	75	835.90	27.96	2.11	3.01	5.55
TMRAH-45-90	0.0324	1.00	45	90	587.24	18.12	2.36	1.90	2.35
TMRUH-45-0	0.0162	1.00	45	0	905.56	55.90	35.61	9.92	13.64
TMRUH-45-15	0.0324	1.00	45	15	2036.50	62.85	50.50	24.30	32.13
TMRUH-45-30	0.0324	1.00	45	30	3260.54	100.63	46.37	35.31	49.26
TMRUH-45-45	0.0266	1.00	45	45	2678.88	100.71	41.68	33.17	39.99
TMRUH-45-60	0.0304	1.00	45	60	1286.95	42.33	6.86	5.47	12.89
TMRUH-45-75	0.0311	1.00	45	75	752.09	24.18	1.97	3.63	4.79
TMRUH-45-90	0.0324	1.00	45	90	608.21	18.77	1.72	1.95	2.32

## Data Availability

The original contributions presented in this study are included in the article. Further inquiries can be directed to the corresponding author.

## References

[1] Lu H., Wang X., Chen T. (2021). In-plane dynamics crushing of a combined auxetic honeycomb with negative Poisson’s ratio and enhanced energy absorption. Thin-Walled Struct..

[2] Luo H.C., Ren X., Zhang Y., Zhang X.Y., Zhang X.G., Luo C., Cheng X., Xie Y.M. (2022). Mechanical properties of foam-filled hexagonal and re-entrant honeycombs under uniaxial compression. Compos. Struct..

[3] Jiang W., Ren X., Wang S.L., Zhang X.G., Zhang X.Y., Luo C., Xie Y.M., Scarpa F., Alderson A., Evans K.E. (2022). Manufacturing, characteristics and applications of auxetic foams: A state-of-the-art review. Compos. Part B Eng..

[4] Han D., Ren X., Luo C., Zhang Y., Zhang X.Y., Zhang X.G., Jiang W., Hao J., Xie Y.M. (2022). Experimental and computational investigations of novel 3D printed square tubular lattice metamaterials with negative Poisson’s ratio. Addit. Manuf..

[5] Zhang J., Lu G., You Z. (2020). Large deformation and energy absorption of additively manufactured auxetic materials and structures: A review. Compos. Part B Eng..

[6] Lu Q., Deng X. (2023). Energy absorption and in-plane mechanical behavior of honeycomb structures with reinforced strut. Compos. Struct..

[7] Zhou J., Zou M., Ng B.F., Ou M. (2025). Emerging sinusoidal structures for energy absorption: Mechanisms, optimizations and applications. Compos. Part B Eng..

[8] Zhang L., Yan S., Liu W., Liu Y., Cai W., Zhang Z., Zhou J. (2025). Mechanical metamaterials with negative Poisson’s ratio: A review. Eng. Struct..

[9] Sengul M., Tasdemir H.A., Tamer A. (2025). Real-world applications of auxetic structures in engineering: A review. Structures.

[10] Yao R., Pang T., Zhang B., Fang J., Li Q., Sun G. (2023). On the crashworthiness of thin-walled multi-cell structures and materials: State of the art and prospects. Thin-Walled Struct..

[11] Andrew J.J., Ubaid J., Hafeez F., Schiffer A., Kumar S. (2019). Impact performance enhancement of honeycombs through additive manufacturing-enabled geometrical tailoring. Int. J. Impact Eng..

[12] Khan N., Riccio A. (2024). A systematic review of design for additive manufacturing of aerospace lattice structures: Current trends and future directions. Prog. Aerosp. Sci..

[13] Jiang D., Thissen H., Hughes T.C., Yang K., Wilson R., Murphy A.B., Nguyen V. (2024). Advances in additive manufacturing of auxetic structures for biomedical applications. Mater. Today Commun..

[14] Nugroho W.T., Dong Y., Pramanik A., Chithirai Pon Selvan M., Zhang Z., Ramakrishna S. (2023). Additive manufacturing of re-entrant structures: Well-tailored structures, unique properties, modelling approaches and real applications. Addit. Manuf..

[15] Donoghue J., Alderson K., Evans K. (2009). The fracture toughness of composite laminates with a negative Poisson’s ratio. Phys. Status Solidi (B).

[16] Choi J., Lakes R. (1996). Fracture toughness of re-entrant foam materials with a negative Poisson’s ratio: Experiment and analysis. Int. J. Fract..

[17] Li T., Liu F., Wang L. (2020). Enhancing indentation and impact resistance in auxetic composite materials. Compos. Part B Eng..

[18] Hu L.L., Zhou M.Z., Deng H. (2019). Dynamic indentation of auxetic and non-auxetic honeycombs under large deformation. Compos. Struct..

[19] Lv W., Li D., Dong L. (2021). Study on blast resistance of a composite sandwich panel with isotropic foam core with negative Poisson’s ratio. Int. J. Mech. Sci..

[20] Ren X., Das R., Tran P., Ngo T., Xie Y. (2018). Auxetic metamaterials and structures: A review. Smart Mater. Struct..

[21] Xing Y., Yang S., Lu S., An Y., Zhao E., Zhai J. (2021). Energy absorption and optimization of Bi-directional corrugated honeycomb aluminum. Compos. Part B Eng..

[22] Wang H., Xiu X., Wang Y., Xue Q., Ju W., Che W., Liao S., Jiang H., Tang M., Long J. (2020). Paper-based composites as a dual-functional material for ultralight broadband radar absorbing honeycombs. Compos. Part B Eng..

[23] Peng C., Tran P. (2020). Bioinspired functionally graded gyroid sandwich panel subjected to impulsive loadings. Compos. Part B Eng..

[24] Huang Z., Zhang X., Yang C. (2020). Experimental and numerical studies on the bending collapse of multi-cell Aluminum/CFRP hybrid tubes. Compos. Part B Eng..

[25] Chen Y., Fu M.-H., Hu H., Xiong J. (2022). Curved inserts in auxetic honeycomb for property enhancement and design flexibility. Compos. Struct..

[26] Xu N., Liu H.-T., An M.-R., Wang L. (2021). Novel 2D star-shaped honeycombs with enhanced effective Young’s modulus and negative Poisson’s ratio. Extrem. Mech. Lett..

[27] Wang H., Zhang Y., Lin W., Qin Q.-H. (2020). A novel two-dimensional mechanical metamaterial with negative Poisson’s ratio. Comput. Mater. Sci..

[28] Deng X., Qin S., Huang J. (2021). Out-of-plane impact analysis for a bioinspired sinusoidal honeycomb. Mech. Adv. Mater. Struct..

[29] Deng X., Liu F., Huang G., Huang J. (2022). Multiobjective optimization for the crashworthiness design of bioinspired sinusoidal honeycombs. Appl. Phys. A Mater. Sci. Process..

[30] Deng X., Qin S. (2023). In-plane energy absorption characteristics and mechanical properties of novel re-entrant honeycombs. Compos. Struct..

[31] Lakes R. (1987). Foam Structures with a Negative Poisson’s Ratio. Science.

[32] Wojciechowski K. (1987). Constant thermodynamic tension Monte Carlo studies of elastic properties of a two-dimensional system of hard cyclic hexamers. Mol. Phys..

[33] Wojciechowski K. (1989). Two-dimensional isotropic system with a negative poisson ratio. Phys. Lett. A.

[34] Evans K.E. (1991). Auxetic polymers: A new range of materials. Endeavour.

[35] Xiao D., Kang X., Li Y., Wu W., Lu J., Zhao G., Fang D. (2019). Insight into the negative Poisson’s ratio effect of metallic auxetic reentrant honeycomb under dynamic compression. Mater. Sci. Eng. A.

[36] Narojczyk J.W., Alderson A., Imre A.R., Scarpa F., Wojciechowski K.W. (2008). Negative Poisson’s ratio behavior in the planar model of asymmetric trimers at zero temperature. J. Non-Cryst. Solids.

[37] Tao Z., Ren X., Zhao A.G., Sun L., Zhang Y., Jiang W., Han D., Zhang X.Y., Xie Y.M. (2022). A novel auxetic acoustic metamaterial plate with tunable bandgap. Int. J. Mech. Sci..

[38] Chen Z., Wu X., Xie Y.M., Wang Z., Zhou S. (2020). Re-entrant auxetic lattices with enhanced stiffness: A numerical study. Int. J. Mech. Sci..

[39] Zhong R., Ren X., Zhang X., Luo C., Zhang Y., Xie Y. (2022). Mechanical properties of concrete composites with auxetic single and layered honeycomb structures. Constr. Build. Mater..

[40] Tabacu S., Stanescu N.D. (2021). A theoretical model for the estimate of the reaction force for 3D auxetic anti-tetra chiral tubular structures under tensile loads. Thin-Walled Struct..

[41] Qi C., Jiang F., Yu C., Yang S. (2019). In-plane crushing response of tetra-chiral honeycombs. Int. J. Impact Eng..

[42] Mizzi L., Attard D., Gatt R., Pozniak A.A., Wojciechowski K.W., Grima J.N. (2015). Influence of translational disorder on the mechanical properties of hexachiral honeycomb systems. Compos. Part B Eng..

[43] Yang W., Huang R., Liu J., Liu J., Huang W. (2022). Ballistic impact responses and failure mechanism of composite double-arrow auxetic structure. Thin-Walled Struct..

[44] Guo M.-F., Yang H., Ma L. (2022). 3D lightweight double arrow-head plate-lattice auxetic structures with enhanced stiffness and energy absorption performance. Compos. Struct..

[45] Grima J., Evans K. (2006). Auxetic behavior from rotating triangles. J. Mater. Sci..

[46] Gibson L., Ashby M., Schajer G., Robertson C. (1982). The mechanics of two-dimensional cellular materials. Proc. R. Soc. Lond. A Math. Phys. Sci..

[47] Zhang J., Lu G., Ruan D., Wang Z. (2018). Tensile behavior of an auxetic structure: Analytical modeling and finite element analysis. Int. J. Mech. Sci..

[48] Zhang J., Lu G., Wang Z., Ruan D., Alomarah A., Durandet Y. (2018). Large deformation of an auxetic structure in tension: Experiments and finite element analysis. Compos. Struct..

[49] Hu L.L., Luo Z.R., Zhang Z.Y., Lian M.K., Huang L.S. (2019). Mechanical property of re-entrant anti-trichiral honeycombs under large deformation. Compos. Part B Eng..

[50] Guo Z., Li Z., Lin J., Mo Z., Li J. (2023). Multi-scale characterization and in-plane crushing behavior of the elliptical anti-chiral honeycomb. Compos. Struct..

[51] Liu K., Cao X., Zhang P., Wu W., Li Y. (2022). Dynamic mechanical performances of enhanced anti-tetra-chiral structure with rolled cross-section ligaments under impact loading. Int. J. Impact Eng..

[52] Dudek K.K., Attard D., Caruana-Gauci R., Wojciechowski K.W., Grima J.N. (2016). Unimode metamaterials exhibiting negative linear compressibility and negative thermal expansion. Smart Mater. Struct..

[53] Dudek K.K., Martinez J.A.I., Ulliac G., Kadic M. (2022). Micro-Scale Auxetic Hierarchical Mechanical Metamaterials for Shape Morphing. Adv. Mater..

[54] Jiang F., Yang S., Ding C., Qi C. (2022). Quasi-static crushing behavior of novel circular double arrowed auxetic honeycombs: Experimental test and numerical simulation. Thin-Walled Struct..

[55] Zhao Y., Deng X., Zheng S., Liu X., Wang Y. (2023). Study on quasi-static axial compression performances and energy absorption of four-star double arrow honeycomb. Compos. Struct..

[56] Wang T., Li Z., Wang L., Zhang X., Ma Z. (2022). In-plane elasticity of a novel arcwall-based double-arrowed auxetic honeycomb design: Energy-based theoretical analysis and simulation. Aerosp. Sci. Technol..

[57] Lan X.-K., Huang Q., Zhou T., Feng S.-S. (2020). Optimal design of a novel cylindrical sandwich panel with double arrow auxetic core under air blast loading. Def. Technol..

[58] Hu L.L., Wu Z.J., Fu M.H. (2018). Mechanical behavior of anti-trichiral honeycombs under lateral crushing. Int. J. Mech. Sci..

[59] Hu L.L., Luo Z.R., Yin Q.Y. (2019). Negative Poisson’s ratio effect of re-entrant anti-trichiral honeycombs under large deformation. Thin-Walled Struct..

[60] Tabacu S., Predoiu P., Negrea R. (2021). A theoretical model for the estimate of plateau force for the crushing process of 3D auxetic anti-tetra chiral structures. Int. J. Mech. Sci..

[61] Zhang C., Zhu Y., Lu F., Wu J., Wu Z. (2023). On the effective elastic constants of anti-tetra chiral tubular structure. Eng. Struct..

[62] Gaspar N., Ren X., Smith C., Grima J., Evans K. (2005). Novel honeycombs with auxetic behaviour. Acta Mater..

[63] Zhu Y., Jiang S., Lu F., Ren X. (2022). A novel enhanced anti-tetra-missing rib auxetic structure with tailorable in-plane mechanical properties. Eng. Struct..

[64] Zhang X.G., Ren X., Jiang W., Zhang X.Y., Luo C., Zhang Y., Xie Y.M. (2022). A novel auxetic chiral lattice composite: Experimental and numerical study. Compos. Struct..

[65] Liu W., Wang X., Hu D., Zhang J., Zhang Q. (2023). Theoretical and numerical study on the in-plane mechanics of an anti-tetrachiral structure. Compos. Struct..

[66] Deng X., Lu Q. (2024). Crushing performance of a novel tetra-missing rib honeycomb: Experimental and numerical studies. J. Mech. Behav. Biomed. Mater..

[67] Chen C., Fang H., Zhu L., Han J., Li X., Qian Z., Zhang X. (2023). Low-velocity impact properties of foam-filled composite lattice sandwich beams: Experimental study and numerical simulation. Compos. Struct..

[68] Xu S., Fu Q., Guang X., Huang H., Deng X. (2025). Performance study of two-ways gradient octagonal hierarchical honeycomb with excellent energy absorption and crashworthiness properties. J. Ind. Inf. Integr..

[69] Sharma D., Hiremath S.S. (2023). Experimental and FEM study on the in-plane and out-plane loaded reversible dual-material bio-inspired lattice structures with improved energy absorption performance. Compos. Struct..

[70] Wang S., Zheng Z., Zhu C., Ding Y., Yu J. (2018). Crushing and densification of rapid prototyping polylactide foam: Meso-structural effect and a statistical constitutive model. Mech. Mater..

[71] Liu H., Zhang E.T., Wang G., Ng B.F. (2022). In-plane crushing behavior and energy absorption of a novel graded honeycomb from hierarchical architecture. Int. J. Mech. Sci..

[72] Zhang X.-C., An C.-C., Shen Z.-F., Wu H.-X., Yang W.-G., Bai J.-P. (2020). Dynamic crushing responses of bio-inspired re-entrant auxetic honeycombs under in-plane impact loading. Mater. Today Commun..

[73] Qi C., Jiang F., Yang S., Remennikov A., Chen S., Ding C. (2022). Dynamic crushing response of novel re-entrant circular auxetic honeycombs: Numerical simulation and theoretical analysis. Aerosp. Sci. Technol..

[74] Cai Z., Deng X. (2025). Energy absorption characteristics of novel hybrid honeycombs under axial crushing. Arch. Civ. Mech. Eng..

[75] Wang H., Lu Z., Yang Z., Li X. (2019). A novel re-entrant auxetic honeycomb with enhanced in-plane impact resistance. Compos. Struct..

